# Needle nephroscope combined with ureteroscope via a single standard percutaneous nephrolithotomy channel for the treatment of complex non-obstructing renal stones

**DOI:** 10.3389/fsurg.2025.1573548

**Published:** 2025-03-25

**Authors:** Xinyu Yi, Jin Li

**Affiliations:** Department of Urology, Xiangtan Central Hospital, Xiangtan, Hunan, China

**Keywords:** needle nephroscope, ureteroscope, combined, standard channel, complex non-obstructing renal stones

## Abstract

**Objective:**

To compare the safety and efficacy of four different surgical approaches for the treatment of complex non-hydronephrotic renal stones.

**Methods:**

A total of 88 patients with complex non-hydronephrotic renal stones, who underwent surgical treatment at Xiangtan Central Hospital from January 2022 to December 2023, were included in this study. The patients were divided into two groups based on their CT values. Group 1 (CT ≥ 1,000) included 22 patients who underwent puncture-assisted single standard percutaneous nephrolithotomy (PCNL) with a laser for stone fragmentation and retrieval (experimental group), and 12 patients who underwent multi-standard percutaneous nephrolithotomy (control group). Group 2 (CT < 1,000) included 21 patients who underwent puncture-assisted single standard PCNL combined with ureteroscopic laser lithotripsy (experimental group), and 33 patients who underwent transurethral ureteroscopic laser lithotripsy (control group). The surgical variables including intraoperative blood loss, operative time, hospital stay, stone clearance rate, and postoperative complications were recorded. Statistical analysis was performed using chi-square test or Fisher's exact test for categorical data, and *t*-test for continuous data.

**Results:**

The two groups were comparable in terms of age, sex, BMI, hypertension, coronary heart disease, diabetes, and preoperative white blood cell count (*P* > 0.01). In both CT ≥ 1,000 and CT < 1,000 groups, the experimental group had significantly less intraoperative blood loss, shorter operative time, and shorter hospital stay compared to the control group (*P* < 0.01). In the CT ≥ 1,000 control group, the stone clearance rate was higher, and two cases of postoperative bleeding (considered arteriovenous fistula) were managed with interventional embolization. In the CT < 1,000 control group, the stone clearance rate was lower, and three cases of postoperative fever (with a maximum temperature of 39.5°C) required an extended antibiotic course for 7 days before discharge.

**Conclusion:**

For complex non-hydronephrotic renal stones, a CT value ≥ 1,000 should be treated with single standard PCNL using a puncture-assisted method; a CT value < 1,000 is better treated with a combination of puncture-assisted single standard PCNL and ureteroscopic laser lithotripsy, with higher safety and efficacy.

## 1 Background

Complex non-hydronephrotic renal stones pose significant challenges in clinical management, particularly when the stones are larger than 2 cm in diameter, multi-focal, or located in difficult-to-reach calyces. Although the need for surgical intervention in asymptomatic calyceal stones remains controversial (1), surgical treatment is necessary for complex stones that cause symptoms (such as pain or infection) or pose a risk to renal function. Percutaneous nephrolithotomy (PCNL) and ureteroscopic laser lithotripsy (URS) are the preferred treatment options for complex renal stones. PCNL is particularly suitable for large or multi-focal stones, while URS is more appropriate for smaller stones or those located in calyces that are difficult to access via PCNL (2). Complex non-hydronephrotic renal stones pose significant challenges in clinical management due to their large size, irregular shape, complex distribution, or staghorn configuration. Although PCNL combined with holmium laser lithotripsy is highly efficient, single-tract PCNL has limitations in clearing stones located in parallel calyces or peripheral areas, resulting in incomplete stone removal. On the other hand, multi-tract PCNL, while effective, is associated with greater trauma and an increased risk of severe complications (3). Flexible ureteroscopy (FURS), with its deflectable fiberoptic bundle allowing up to 275°/185° of upward/downward deflection and both active and passive bending capabilities, can access all calyces and plays a crucial role in the treatment of renal stones (4). However, the efficiency of holmium laser lithotripsy under FURS is low for hard stones, often necessitating staged procedures. To address these challenges, we employed a combined approach using needle-perc nephroscopy and FURS through a single standard percutaneous renal tract for the treatment of complex non-hydronephrotic renal stones. This minimally invasive technique enhances the safety and efficacy of stone removal, providing a reliable surgical option for patients with complex non-hydronephrotic renal stones.

## 2 Materials and methods

Patients who underwent surgery for complex non-hydronephrotic renal stones at Xiangtan Central Hospital from January 2022 to December 2023 were enrolled. Inclusion criteria: (1) Diagnosis confirmed by ultrasound or CT and meeting the “EUA 2022 Guidelines on Diagnosis and Treatment of Renal Stones” for complex non-hydronephrotic renal stones. (2) Presence of symptoms (such as pain or infection) or risk to renal function due to complex renal stones, with indications for PCNL or URS. (3) Indications for percutaneous nephrolithotomy (PCNL) or ureteroscopy with holmium laser lithotripsy. (4) Underwent surgery and had complete clinical data. (5) Ethical approval was obtained, and the patient consented to participate in the study. Exclusion criteria: (1) Coagulation disorders or anticoagulant therapy within two weeks prior to surgery. (2) Congenital urological malformations like scoliosis or ureteral stenosis. (3) Severe diseases like hydronephrosis, malignant kidney tumors, or systemic diseases. (4) Pregnant or lactating women (5) Mental illness.

### 2.1 Definition of complex non-hydronephrotic renal stones

These are stones greater than 2 cm in diameter, multi-focal, or located in difficult-to-reach renal calyces, but without causing obvious hydronephrosis. The term “difficult-to-reach” refers to stones located in calyces with steep infundibulopelvic angles (IPA > 45°), narrow or long calyceal infundibula (<5 mm in diameter or >3 cm in length), or stones in calyces with complex anatomy that makes access challenging during standard PCNL or ureteroscopy. The diagnosis of non-hydronephrotic kidneys was confirmed by preoperative ultrasound and CT scans. Specifically, non-hydronephrosis was defined as the absence of significant dilation of the renal pelvis and calyces, with normal renal parenchymal thickness on ultrasound. On CT scans, non-hydronephrosis was characterized by the absence of significant dilation of the renal pelvis and calyces, as well as no obstruction at the ureteropelvic junction (UPJ). These criteria are consistent with the “EAU 2022 Guidelines on Diagnosis and Treatment of Renal Stones” The definition reflects complex factors such as size, location, number, and composition of the stones, while non-hydronephrosis indicates that the urinary tract is not completely obstructed and renal function has not been severely damaged (1).

### 2.2 Grouping

Patients were divided into two groups based on their CT values: CT ≥ 1,000 HU: 34 cases, randomized into experimental group (22 cases, puncture-assisted single-channel PCNL) and control group (12 cases, multi-channel PCNL). CT < 1,000 HU: 54 cases, randomized into experimental group (21 cases,puncture-assisted single standard PCNL combined with ureteroscopic laser lithotripsy) and control group (33 cases, transurethral ureteroscopy).

### 2.3 PCNL access and tract details:needle nephroscope group

A standard 24 Fr PCNL tract was established to ensure sufficient working space and effective stone clearance. The needle nephroscope was introduced through this tract for stone fragmentation and retrieval.

### 2.4 Multi-channel PCNL group

Standard 24 Fr PCNL tracts were established, and multiple tracts (typically 2–3) were created as needed based on stone size, location, and complexity. The number of tracts was determined intraoperatively to ensure complete stone clearance while minimizing renal trauma.

### 2.5 Surgical procedure for needle PCNL

#### 2.5.1 Patient positioning and anesthesia

Patients were placed in the oblique supine lithotomy position under general anesthesia. This position allows simultaneous access to the urethra and the flank, facilitating both retrograde ureteroscopy and percutaneous nephrolithotomy.

#### 2.5.2 Ureteral access and retrograde pyelography

A ureteral access sheath was placed under fluoroscopic guidance, and retrograde pyelography was performed to delineate the renal anatomy and identify the target calyx for puncture.

#### 2.5.3 Puncture and tract establishment

Under fluoroscopic and ureteroscopic guidance, a percutaneous puncture was made into the target calyx using an 18-gauge needle. The puncture site was carefully selected to minimize the angle between the tract and the target calyx, especially for lower pole stones. A guidewire was then advanced through the needle into the renal pelvis**.**

#### 2.5.4 Tract dilation and sheath placement

The tract was dilated using a serial dilator system, and a 16–18 Fr nephrostomy sheath was placed to establish the working channel for the needle nephroscope**.**

#### 2.5.5 Stone fragmentation and retrieval

The needle nephroscope was introduced through the sheath, and stones were fragmented using a holmium:YAG laser (365 µm fiber, 0.8–1.2 J, 10–15 Hz). Stone fragments were retrieved using a basket or suction device. For stones located in parallel calyces or difficult-to-reach areas, a flexible ureteroscope was introduced through the same tract to assist in stone clearance.

#### 2.5.6 Postoperative management

**Figure 1:**
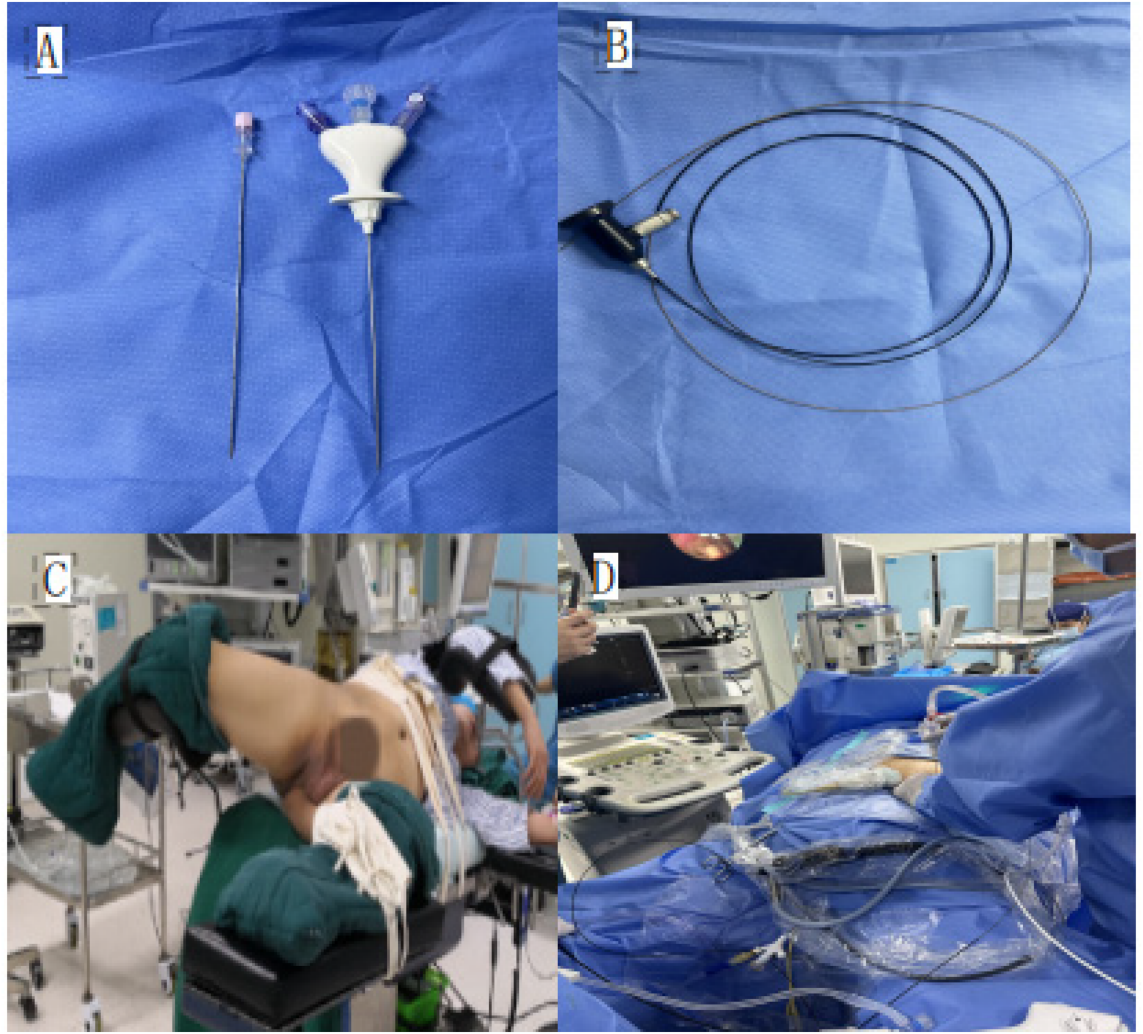
**(A)** Puncture-assisted nephroscope sheath; **(B)** puncture-assisted nephroscope; **(C)** oblique supine lithotomy position; **(D)** puncture-assisted nephroscope combined with ureteroscopic stone removal surgery.

A nephrostomy tube was placed at the end of the procedure, and its position was confirmed by fluoroscopy. The tube was typically removed 24–48 h postoperatively if no significant bleeding or infection was observed.All surgeries were performed by urologists with extensive experience in percutaneous nephroscopy and ureteroscopy. The puncture-assisted nephroscope, percutaneous nephrostomy sheath, and surgical positioning are shown in Figure 1.

The size of the stones was measured using non-contrast computed tomography (NCCT) with 5 mm slice axial images, and the stone diameter was recorded. Fragments with a diameter ≤2 mm were considered as no residual stones. Postoperative complications were assessed using the Clavien-Dindo grading system. (Grade I represents minor complications that do not require special treatment; Grade II requires drug treatment (e.g., antibiotics or blood transfusion); Grade III requires invasive interventions, divided into IIIa (local anesthesia or no anesthesia) and IIIb (general anesthesia); Grade IV represents life-threatening complications requiring ICU treatment, divided into IVa (single organ failure) and IVb (multiple organ failure); Grade V represents patient death). Surgical parameters, including intraoperative blood loss, surgical and hospital stay durations, renal function, stone clearance rate, and perioperative complication rate, were recorded.

Descriptive statistics were conducted using SPSS 26.0 to explore the four different surgical methods for treating complex non-obstructing renal stones. Scatter plots were generated using R (4.3.2) software, and comparisons of categorical data between groups were made using the chi-square test or Fisher's exact test. *P* ≤ 0.01 (two-sided) was considered statistically significant.

## 3 Results

A total of 88 patients did not observe significant differences in comparison of general data such as gender, age, BMI, underlying condition, and preoperative leukocytes, the most frequently selected puncture site was the lower pole calyx in the Experimental Group (Table 1).

**Table 1:** Basic characteristics of the 4 groups of non-obstructing renal stones (*n* = 88).

Characteristics	Ct ≥ 1,000		*p*	Ct < 1,000		*p*
	Experimental group	Control group		Experimental group	Control group	
Number of patients (no)	22	12		21	33	
Age (years)	47.23 ± 7.32	46.77 ± 6.97	0.82	48.25 ± 7.10	47.44 ± 5.10	0.76
Gender (no)			0.69			0.67
Male	10	8		11	15	
Female	12	4		10	18	
BMI (kg/m^2^)	23.17 ± 4.18	23.19 ± 3.37	0.85	22.19 ± 4.08	23.15 ± 3.89	0.95
Hypertension (no)	3	4	0.24	2	2	0.27
Coronary heart disease (no)	2	1	0.74	3	1	0.78
Diabetes (no)	2	0	0.53	3	2	0.63
Preoperative leukocyte count (× 10⁹/L)	8.61 ± 3.62	8.67 ± 3.92	0.75	7.61 ± 2.62	7.21 ± 3.45	0.71
Puncture site (no)						
Lower pole calyx	13 (59%)			16 (76%)		
Middle pole calyx	6 (27%)			3 (14%)		
Upper pole calyx	3 (14%)			2 (10%)		

Multivariate logistic regression analysis showed that surgical outcomes, including intraoperative blood loss, surgical and hospital stay durations, renal function, stone clearance rate, and perioperative complication rates, were associated with the type of surgery performed (see Table 2).

**Table 2:** Four different surgical methods for treating Complex Non-obstructing renal stones.

Outcomes	Ct ≥ 1,000		*p*	Ct < 1,000		*p*
	Experimental group	Control group		Experimental group	Control group	
Intraoperative blood loss (ml)	22.11 ± 1.81	41.38 ± 6.20	<0.00	22.18 ± 2.51	13.18 ± 3.11	<0.00
Surgical time (min)	61.38 ± 6.20	72.43 ± 9.36	<0.00	68.25 ± 7.10	87.44 ± 5.10	<0.00
Hospitalization time (d)	9 ± 3	13 ± 2	<0.00	6 ± 3	7 ± 1	<0.00
Stone clearance rate (%)	94%	97%	<0.00	95%	88%	<0.00
Complications rate (%)	9.09% (2 cases Grade I)	25% (1 case Grade I + 2 cases Grade III)	<0.00	19.05% (4 cases Grade I)	22.81% (5 cases Grade I + 3 cases Grade II)	<0.00

Additionally, we found that regardless of whether the CT value was ≥1,000 or <1,000, the experimental group had significantly lower intraoperative blood loss, shorter surgery time, and shorter hospital stay compared to the control group (*P* < 0.00). In the CT ≥ 1,000 control group, the stone clearance rate was higher than that of the experimental group, with two cases of postoperative bleeding suspected to be due to an arterial-venous fistula, which were treated with interventional embolization. In the CT < 1,000 control group, the stone clearance rate was lower than the experimental group, and three cases of postoperative chills and fever (with a maximum temperature of 39.5°C) occurred. The postoperative antibiotic course was extended to 7 days before discharge.

**Figure 2:**
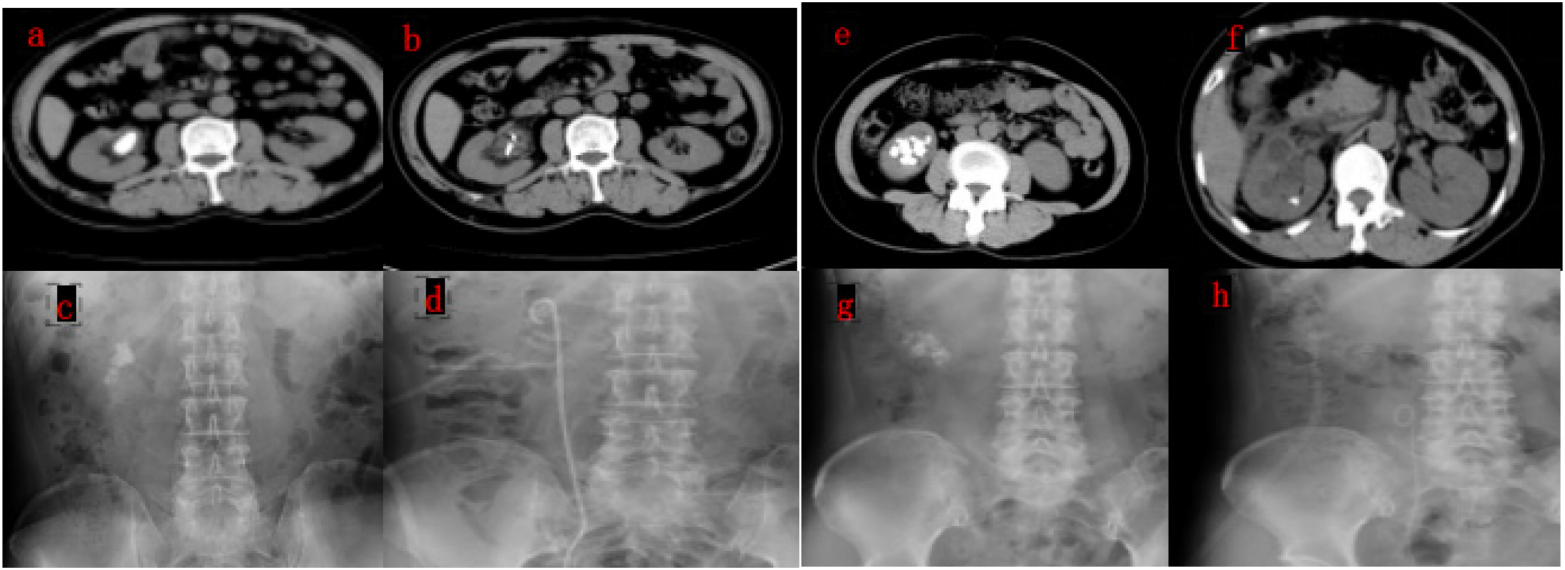
Representative preoperative and postoperative CT images of patients in the experimental and control groups.(**(a,b)** preoperative and postoperative CT images of a patient undergoing puncture-assisted single standard PCNL with needle nephroscope, showing complete stone clearance. **(c,d)** Preoperative and postoperative x-ray images of the same patient, demonstrating the effectiveness of the procedure. **(e,f)** Preoperative and postoperative CT images of a patient undergoing puncture-assisted single standard PCNL combined with flexible ureteroscopy, showing residual stones in the lower pole calyx. **(g,h)** Preoperative and postoperative x-ray images of the same patient, illustrating the stone distribution and postoperative outcomes.).

Figure 2 shows representative preoperative and postoperative CT and X-ray images of patients in the experimental and control groups, demonstrating the effectiveness of the puncture-assisted single standard PCNL with needle nephroscope and the combined approach of puncture-assisted single standard PCNL and flexible ureteroscopy.

## 4 Discussion

Since the introduction of extracorporeal shock wave lithotripsy (SWL), percutaneous nephrolithotomy (PCNL), ureterorenoscopy (URS), and retrograde intrarenal surgery (RIRS) in the 1980s, the treatment of renal stones has undergone a profound transformation (5, 6). The success of these minimally invasive procedures has made open surgery for urinary stones rare. However, in the case of recurrent non-obstructing renal stones, due to the thickness of renal parenchyma, the relatively narrow renal pelvis and calyces, small operative space, and the higher likelihood of intraoperative bleeding, these stones present significant challenges. Additionally, puncture technique is demanding, and difficulties in guidewire placement or its displacement are not uncommon. Therefore, selecting an appropriate treatment method from these minimally invasive approaches remains a controversial issue.

The choice between miniaturized PCNL and RIRS for the treatment of renal stones has been widely debated in recent years. RIRS has been shown to offer several advantages, including the highest stone-free rates (SFR), lower complication rates, shorter operative times, and shorter hospital stays compared to miniaturized PCNL (7, 8). These benefits make RIRS an attractive option, particularly for smaller stones or those located in difficult-to-access calyces. However, for larger stones (>2 cm), RIRS may require prolonged surgical times, increasing the risk of postoperative infections and necessitating staged procedures (9).

On the other hand, miniaturized PCNL, including needle PCNL, has its own advantages. It is particularly effective for larger stones and offers a more cost-effective approach compared to RIRS, especially in settings where repeated RIRS procedures may be required (10). Additionally, miniaturized PCNL allows for the use of negative pressure suction devices, which reduce renal pelvic pressure and expedite stone removal. However, it is associated with a higher risk of renal injury and bleeding due to the need for multiple tracts in some cases (10).

In our study, we combined the advantages of miniaturized PCNL and RIRS by using a puncture-assisted single standard PCNL tract combined with ureteroscopic laser lithotripsy. This approach demonstrated superior stone clearance rates and fewer postoperative infections compared to RIRS alone, particularly for stones with a CT value < 1,000 HU. Our findings align with previous studies suggesting that a combined approach may offer the best of both worlds, especially for complex stones that are difficult to treat with a single modality (9).

The use of FURS via the PCNL tract has been proposed as an alternative to retrograde FURS during PCNL. One of the main advantages of FURS via the PCNL tract is the ability to access stones in calyces that are difficult to reach through a retrograde approach. However, limitations of this technique include flexion limitation, especially when the angle between the PCNL tract and the target calyx is acute, and difficult maneuverability of the ureteroscope within the narrow PCNL tract.To overcome these limitations, we adjusted the puncture path under the guidance of the ureteroscope, ensuring optimal access to the target calyx. Despite these adjustments, some residual stones were observed in calyces with acute angles, especially in the lower pole. In our study, the majority of residual stones in the experimental group were located in the lower pole calyces, where the angle between the PCNL tract and the calyx was most acute. This highlights the importance of precise puncture planning and the potential need for additional techniques, such as flexible ureteroscopy, to address these challenging cases (11).

Retrograde FURS offers greater flexibility and maneuverability, allowing access to all calyces, including those with acute angles. However, retrograde FURS may require longer operative times and is less effective for larger stones, particularly those >2 cm in diameter. In our control group (CT < 1,000), where retrograde FURS was used, the stone clearance rate was lower compared to the experimental group, and residual stones were more frequently observed in the lower pole calyces. This suggests that while retrograde FURS is effective for smaller stones, it may not be sufficient for complex stones located in difficult-to-access calyces (12).

Furthermore, it is important to note that RIRS is associated with a potential risk of procedure-related infections, particularly in cases where prolonged operative times are required. In our study, the experimental group (Group 2) demonstrated a significantly lower incidence of infection-related events compared to the control group. This can be attributed to the combined approach of needle PCNL and FURS, which reduces the operative time and minimizes the risk of bacterial translocation. The use of negative pressure suction during PCNL also helps to lower renal pelvic pressure, further reducing the risk of postoperative infections. These findings highlight the safety and efficacy of our combined approach in minimizing infection-related complications, which is a critical consideration in the management of complex renal stones (2).

The needle-shaped nephroscope was invented in 2019 and is currently the smallest nephroscope in the world. It was initially used for stones with a diameter of less than 1.5 cm (13). Similar to micro-channel PCNL, the needle-shaped nephroscope is used under the guidance of a ureteroscope to establish a channel. The advantage is that, under the ureteroscope's vision, the puncture path of the needle-shaped nephroscope can be adjusted to ensure precise stone fragmentation. Moreover, after stone fragmentation with the needle-shaped nephroscope, a ureteroscope can be used to clear stone fragments and irrigate the renal pelvis, which reduces renal pelvic pressure. Studies have demonstrated (14–16) that micro-channel PCNL is safe and effective for fragmenting lower calyceal stones.

The size and number of PCNL renal access tracts have a significant impact on surgical complications and stone-free rates. Larger tracts facilitate the use of negative pressure suction devices, which reduce renal pelvic pressure and expedite stone removal. However, larger tracts also increase the risk of surgical complications (17, 18). This aligns with our study's results, where in the Ct ≥ 1,000 group, the experimental group had significantly less intraoperative blood loss, shorter surgical times, and shorter hospital stays compared to the control group. The reason for this is that multi-channel PCNL requires multiple tracts to be established for stone extraction, often requiring significant manipulation to locate stones, which increases the risk of damage to surrounding renal vessels and organs, thereby raising postoperative complications. In contrast, using the needle-shaped nephroscope to establish a single standard percutaneous renal access tract with laser lithotripsy enables precise stone localization without extensive manipulation. Although the stone clearance rate is somewhat reduced, this approach significantly lowers the risk of renal injury and reduces complications.

When Ct < 1,000, the hardness of the stones is relatively lower. With the widespread use of negative pressure suction sheaths, most urologists opt for ureterorenoscopy (URS) for stone fragmentation. Some studies have indicated that RIRS offers comparable stone clearance rates to PCNL and has a higher safety profile (19). However, for stones larger than 2 cm, RIRS can result in prolonged surgical times, increasing the risk of postoperative infections. After comprehensive consideration, we chose to combine the needle-shaped nephroscope with RIRS. For stones with a Ct value < 1,000, this combination demonstrated superior stone clearance rates and fewer postoperative infections compared to using RIRS alone.

However, our study has several limitations. The sample is limited to patients from Xiangtan Central Hospital, and the surgical outcomes may vary depending on the surgeon's experience. Despite two experienced radiologists performing the CT value measurements to minimize errors caused by including adjacent renal parenchyma or stones, a small number of patients with fatty tissue or chronic infections might have led to inaccurate measurements. A larger and more diverse sample is needed for further validation.Additionally, our study did not include a direct comparison between (A) needle PCNL plus FURS, (B) needle PCNL only, and (C) RIRS only. This limitation is due to the study design, which was based on CT value grouping and the need to maintain a manageable sample size. While such a comparison would provide valuable insights into the relative efficacy and safety of these approaches, it was beyond the scope of the current study. Future studies with a larger and more diverse sample are needed to explore this important question.

## 5 Conclusion

For complex non-hydronephrotic renal stones, a CT value ≥ 1,000 should be treated with single standard PCNL using a puncture-assisted method; for CT values < 1,000, a combination of puncture-assisted single standard PCNL and ureteroscopic laser lithotripsy offers higher safety and efficacy.

## Data Availability

The raw data supporting the conclusions of this article will be made available by the authors, without undue reservation.
